# Hearing preservation outcomes with motorized cochlear implant electrode insertion: matched-cohort observations

**DOI:** 10.3389/fsurg.2025.1700744

**Published:** 2025-11-07

**Authors:** Yannik Oetiker, Philipp Aebischer, Marco Caversaccio, Georgios Mantokoudis, Stefan Weder

**Affiliations:** 1 Department of Ear, Nose and Throat Diseases, Head and Neck Surgery, Inselspital, Bern University Hospital, University of Bern, Bern, Switzerland; 2 Hearing Research Laboratory, ARTORG Center for Biomedical Engineering Research, University of Bern, Bern, Switzerland

**Keywords:** cochlear implantation, hearing preservation, motorized insertion tool (MIT), robotic-assisted surgery, speech comprehension, patient-reported outcome measures (PROMs)

## Abstract

**Background:**

Cochlear implants (CIs) are an established treatment for severe sensorineural hearing loss and are increasingly used in patients with substantial residual hearing. Preservation of residual hearing is associated with improved outcomes, including speech understanding in noise, natural sound perception, and spatial hearing. Manual electrode insertion, however, is limited by hand tremor and abrupt maneuvers, which can cause intracochlear trauma. Motorized insertion tools (MITs) have been developed to enable slow, continuous, and highly controlled electrode advancement.

**Methods:**

We conducted the first clinical evaluation of the OTOARM/OTODRIVE MIT system. Twenty-six patients underwent implantation with lateral wall electrodes using MIT and were compared with a matched retrospective cohort who received manual insertion. Surgical workflow integration, electrode positioning, residual hearing, speech comprehension, and patient-reported outcome measures (PROMs) were assessed at 1 and 6 months postoperatively.

**Results:**

MIT integration into the surgical routine was feasible without major workflow disruptions. Angular insertion depth and electrode positioning did not differ significantly between groups. Patients with favorable preoperative hearing showed slightly better postoperative low-frequency pure tone thresholds in the MIT group, although statistical significance was not reached. Speech comprehension outcomes were comparable between groups. PROMs indicated greater gains in several subscales for the MIT-assisted cohort, despite incomplete data and limited statistical power.

**Conclusion:**

MIT-assisted cochlear implantation was feasible and demonstrated a tendency toward improved hearing preservation and subjective benefit. However, the small sample size, retrospective controls, and incomplete PROM data limit definitive conclusions. Larger, blinded and randomized trials are needed to determine the clinical value of MIT systems for both objective and patient-reported outcomes.

## 1 Introduction

Patients with severe sensorineural hearing loss have benefited significantly from cochlear implants (CIs), which are an established treatment modality for restoring speech comprehension and improving quality of life (1–3). In recent years, CIs have been increasingly used in individuals with substantial residual hearing (4, 5). Although this residual hearing is typically insufficient for effective speech understanding without an implant, preserving it during and after cochlear implantation has been associated with improved patient outcomes (6). This preservation enables an *enhanced electric hearing*, in which patients benefit from both the electrical stimulation provided by the CI and their remaining residual acoustic hearing. Improved outcomes have been reported in several domains, including better speech understanding in noisy environments, more natural sound perception, and enhanced spatial hearing (7–9).

To support preservation of residual hearing, the concept of *soft surgery* has gained increasing relevance (10–12). The aim of this surgical technique is to minimize trauma to inner ear structures during electrode insertion, thereby maintaining cochlear integrity and residual function (13, 14). This surgical approach was first described several years ago (15, 16). However, more recent studies have shown that manual insertion, even when performed by experienced surgeons, presents specific limitations (17–21). In particular, the final millimeters before the electrode reaches its target position are critical: this phase is susceptible to hand tremors and abrupt surgical maneuvers, resulting in significant force and pressure peaks within the cochlea (22–24).

To address these limitations, systems for “robotic-assisted electrode insertion” have been developed (25–27). These systems typically consist of a motorized forcep equipped with a foot-pedal-controlled advancement mechanism, allowing for slow, continuous, and highly controlled electrode advancement with precise monitoring of insertion depth (28–30). In the present manuscript, we refer to this system as a motorized insertion tool (MIT). The effectiveness of MIT has been demonstrated in a temporal bone model with human cochlear properties and integrated multisensory measurements, where its use significantly reduced intracochlear pressure and insertion-related force peaks (27, 31). These findings were corroborated by a cadaveric study conducted by Claussen et al., which reported lower trauma scores for MIT-assisted insertions (32).

It has further been demonstrated that MIT systems can be integrated into the clinical workflow without substantial additional effort (33–37). Table 1 provides an overview of clinical applications of different MIT systems. Initial clinical results suggest that patients implanted using MIT technology may experience improved preservation of functional residual hearing at low frequencies (38, 39). In terms of structural hearing preservation, early evidence also points to advantages, with fewer electrode translocations into the scala vestibuli reported for MIT-assisted procedures (33, 40, 41). Findings regarding speech comprehension are less consistent. While some studies observed similar speech discrimination outcomes following MIT-assisted insertion, others reported marked improvements (21, 42–44).

**Table 1:** Clinical publications on motorized insertion tools for CI implantation.

Study (Year)	n (Robotic)	Control group	Robotic system	Primary outcome	Key findings
Claussen et al. (32)	6	None	iotaSOFT${}^{\mathrm{TM}}$	Feasibility, trauma monitoring (ECochG)	Slower insertion speeds allow for more precise control of electrode insertion
Gersdorff et al. (44)	24	15	RobOtol${}^{®}$	Impedance, low-frequency hearing, speech scores	MIT-assisted insertion leads to better auditory performance and more atraumatic electrode insertion
Khan et al. (43)	27	24	iota-SOFT	Hearing preservation, speech-in-noise	Improved hearing preservation in MIT-assisted cohort 1 year postoperative

Despite promising developments, the clinical validation of MIT systems remains limited, and particularly, their subjective benefits have not been extensively investigated (43, 44). The present study pursued three primary objectives. First, we aimed to contribute to the growing body of clinical data on CI insertions using MIT systems. Second, we sought to present the first clinical results obtained with the OTOARM/OTODRIVE system. Third, in addition to objective audiological data, such as functional hearing preservation and speech perception, we assessed subjective outcomes using patient-reported outcome measures (PROMs). Employing a matched-cohort design, we compared the outcomes of manual insertion with those of MIT-assisted insertion. Our clinical evaluation encompassed surgical workflow integration, insertion depth, preservation of residual hearing, speech perception, and patient satisfaction. We hypothesized that the use of MIT would lead to reduced postoperative hearing loss and enhanced patient satisfaction and speech understanding.

## 2 Methodology

### 2.1 Study design and patient selection

This study adhered to the local ethics committee guidelines as specified in the decision (BASEC ID 2025-00363). Patients provided their written informed consent. Twenty-six patients who were implanted with a lateral wall electrode (MED-EL, Austria) using the OTOARM/OTODRIVE system (CASCINATION AG, Switzerland & MED-EL, Austria) were prospectively enrolled. These 26 cases were compared with a retrospectively matched cohort. Manual cases were matched to the 26 MIT-assisted cases by an investigator who, in a first step, had access only to preoperative data. Matching was based on age (±15 years), sex, electrode model, and preoperative low-frequency PTA (±10 dB). The investigator was blinded to all postoperative outcomes during the matching process. Only cases performed from November 2020 onward were included to ensure that the same two surgeons conducted all procedures in both the MIT-assisted and matched manual cohorts. This approach was intended to minimize potential confounding related to baseline hearing status, electrode characteristics, or surgical experience. Patients with a history of previous ear surgery or atypical cochlear anatomy, such as malformations, were excluded.

### 2.2 Device and surgery

**Figure 1:**
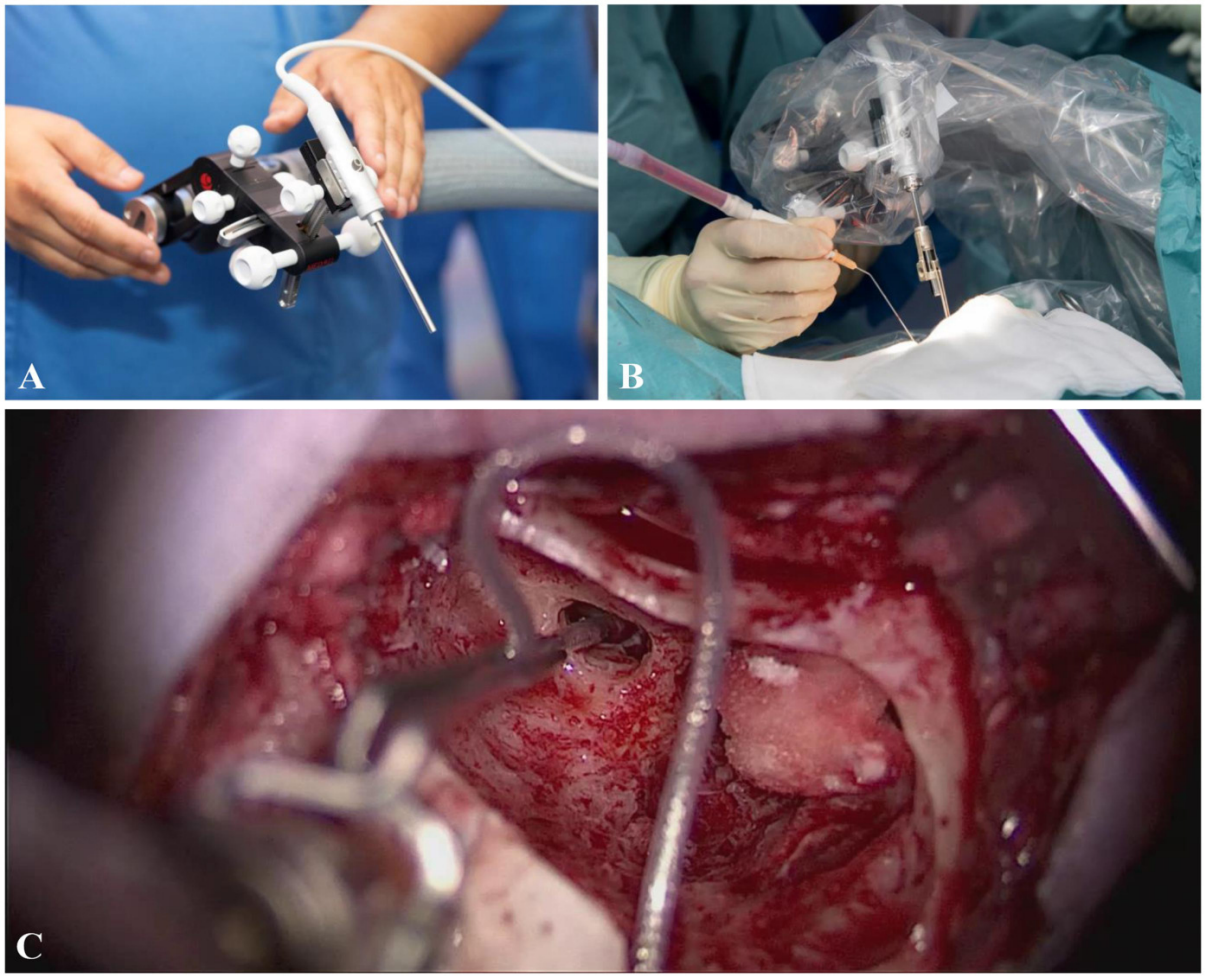
**(A)** Before draping, the OTOARM/OTODRIVE system is positioned to allow proper alignment of the motorized forceps. **(B)** The system is sterilely draped, and the forceps holder is attached. After identification of the round window, the forceps are aligned with the trajectory of the basal turn. **(C)** The electrode is advanced to the round window using the screw and motorized drive, then inserted at a feed rate of 0.1 mm/s via foot pedal control.

Manual and MIT insertions were performed by two experienced surgeons. Manual cases were performed according to the established soft surgery technique (15, 45), while MIT-assisted insertions followed the manufacturer’s recommendations and guidelines (CASCINATION AG, Switzerland and MED-EL, Austria). The surgical approach in both techniques (manual and MIT) was identical up to the identification of the round window. The OTOARM/OTODRIVE system consists of a flexible arm, a manual alignment unit, and a motorized drive unit that enables controlled advancement of the electrode (Figure 1). Subsequently, in the MIT cases, the OTOARM was positioned and aligned so that the insertion trajectory matched the basal turn of the cochlea. The OTODRIVE unit was then retracted, and the CI electrode was secured in the motorized forceps. After opening the round window membrane, using the tool-axis knob of the OTOARM aligner, the electrode was manually advanced until the tip reached the round window. Then, the insertion was continued at the minimal feed rate of 0.1 mm/s, advancing the electrode into the cochlea using foot pedals.

### 2.3 Data collection

Approximately four weeks after surgery, high-resolution temporal bone computed tomography (CT) was acquired. OtoPlan${}^{®}$ software (CASCINATION AG, Switzerland) was used sequentially to perform automated segmentation of the cochlear structures. The angular insertion depths of the most apical electrode contact (E1) and the most basal electrode contact (E12) were calculated relative to the center of the round window. Angles were measured from the midmodiolar axis to each electrode contact along the cochlear spiral, following the method described by Heutink et al. (46).

For the assessment of residual hearing and speech perception with the implant, patients were evaluated according to the institutional protocol, which included pure-tone audiometry (125–8,000 Hz) conducted preoperatively and at 1 and 6 months postoperatively. The relative change in the pure-tone audiogram threshold was calculated with reference to a maximum of 120 dB and normalized to the preoperative measurement.

In addition, the Freiburg speech comprehension test for mono- and disyllabic words was administered at 65 dB before implantation and at 1, 3, and 6 months postoperatively.

Patient-reported outcome measures (PROMs) were obtained using the Speech, Spatial, and Qualities of Hearing Scale (SSQ-12), a validated 12-item questionnaire capturing subjective hearing performance in everyday listening situations (47). The SSQ-12 was administered preoperatively and at the 6-month postoperative follow-up to evaluate changes in speech perception, spatial hearing, and sound quality. Scores were analyzed descriptively for nine subscales, with the total score defined as the mean of all subscale scores (48). Administration was limited to patients for whom a validated version in German, French, or Italian was available. As PROMs had only recently been incorporated into the institutional assessment protocol, data were incomplete for some individuals in the retrospective manual cohort.

### 2.4 Data analysis

Data distribution was assessed using the Shapiro–Wilk test. As the data in our cohort did not follow a normal distribution, non-parametric statistical tests were employed for the analysis using the Wilcoxon signed-rank test in Python 3.12. Statistical comparisons were conducted for relative hearing threshold levels, functional low-frequency hearing levels, Freiburg speech test results, angular insertion depths, and SSQ-12 scores using NumPy. P-values <0.05 were considered statistically significant. No specific measures were applied to adjust for potential confounding factors. Figures were created with Matplotlib and Seaborn.

## 3 Results

The median age at implantation was 55 years in the robotic group and 59 years in the manual group. Implanted side and etiology of hearing loss were comparable, with progressive hearing loss of unknown origin being the most frequent cause in both cohorts. Identical electrode models were used across groups: 18 patients received Flex28 arrays, 7 received FlexSoft arrays, and 1 received a Flex26 array.

### 3.1 Operative time and electrode insertion metrics

No statistical differences in total surgical duration were observed between the two groups, with a median duration of 106 min (IQR=39) in the manual group and 114 min (IQR=27) in the MIT group. The position of the most basal contact (E12) relative to the round window was comparable between groups, with the MIT-assisted cohort demonstrating a median angular position of $16^{\circ }$ ($\text{IQR}=22^{\circ }$) and the manual cohort a median of $15^{\circ }$ ($\text{IQR}=19^{\circ }$). In 3 MIT-assisted cases and 2 manual cases, the basal contact was located proximally to the round window plane, reaching a maximum offset of $-2^{\circ }$ in both cohorts. For the most apical contact (E1), the median angular insertion depth was $574^{\circ }$ ($\text{IQR}=72^{\circ }$) in the MIT-assisted group and $552^{\circ }$ ($\text{IQR}=79^{\circ }$) in the manual group, indicating similar overall insertion depths between approaches (p=0.08). Variability in insertion angles was marginally lower in the MIT-assisted group with an IQR of $71.5^{\circ }$ vs. $78.6^{\circ }$ in the manual group.

### 3.2 Audiometric results

Postoperative changes in hearing were evaluated by stratifying patients into three subgroups according to their preoperative low-frequency pure tone average (LFPTA), representing baseline functional residual hearing. The subgroups were defined as LFPTA ≤60dB, 61−80dB, and >80dB to allow assessment of hearing preservation relative to initial auditory capacity. Negative values indicate that hearing thresholds worsened after surgery, with larger negative values reflecting greater loss in low-frequency hearing.

**Figure 2:**
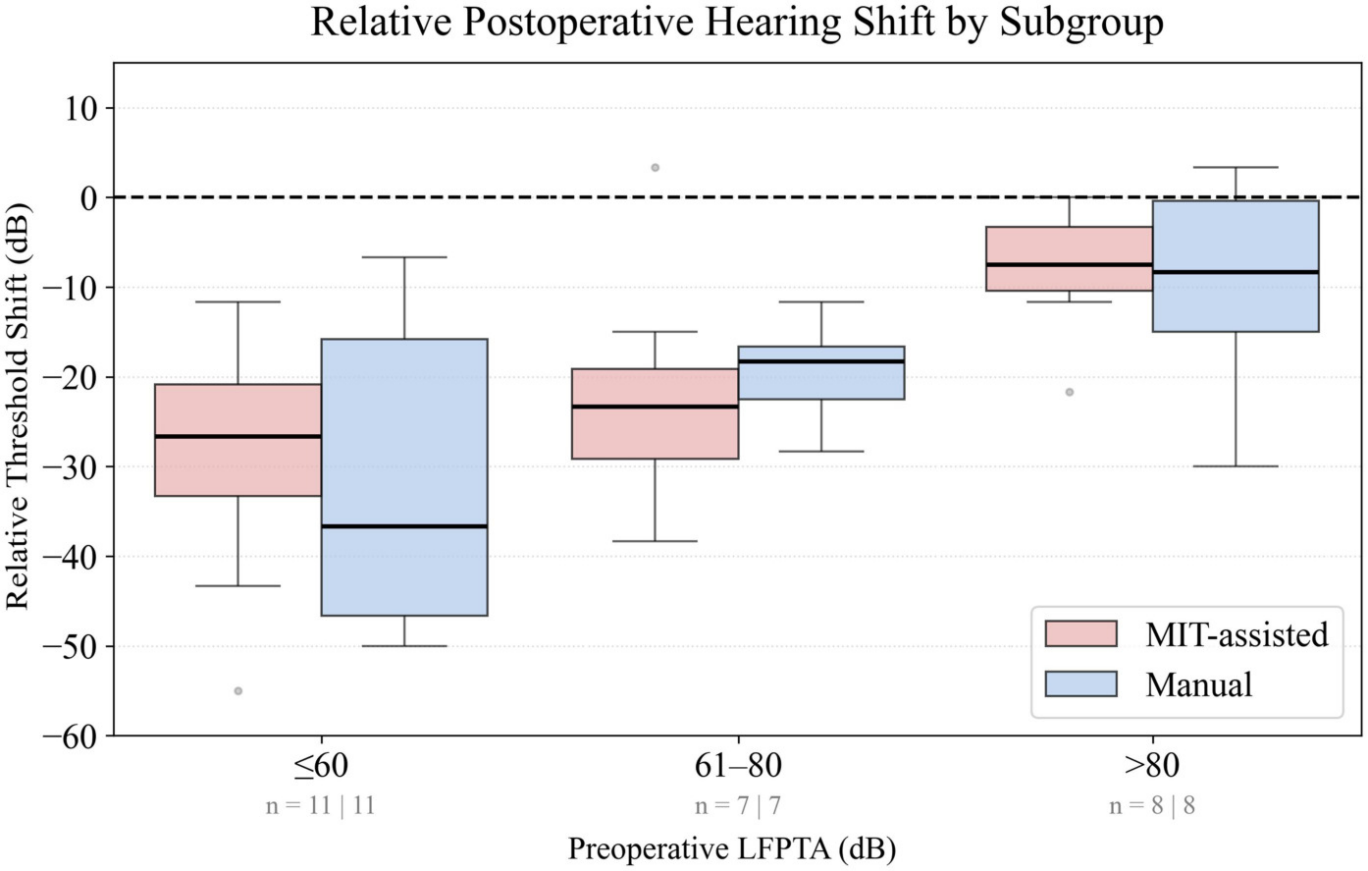
Boxplots display the relative threshold shift (dB) from preoperative to 1-month postoperative low-frequency pure-tone average (LFPTA; 125–500 Hz), calculated with reference to a 120 dB ceiling. Patients were stratified into three subgroups according to preoperative LFPTA: 0–60 dB, 61–80 dB, and >80 dB. Threshold shifts were compared between MIT-assisted (red) and manual (blue) cochlear implantations. Negative values indicate deterioration of hearing. Boxes represent the interquartile range (IQR), horizontal lines denote the median, and whiskers extend to 1.5× the IQR. Outliers are shown as individual gray points. The dashed line at 0 dB marks no threshold change. Patient numbers per cohort in each subgroup are indicated below the x-axis.

**Figure 3:**
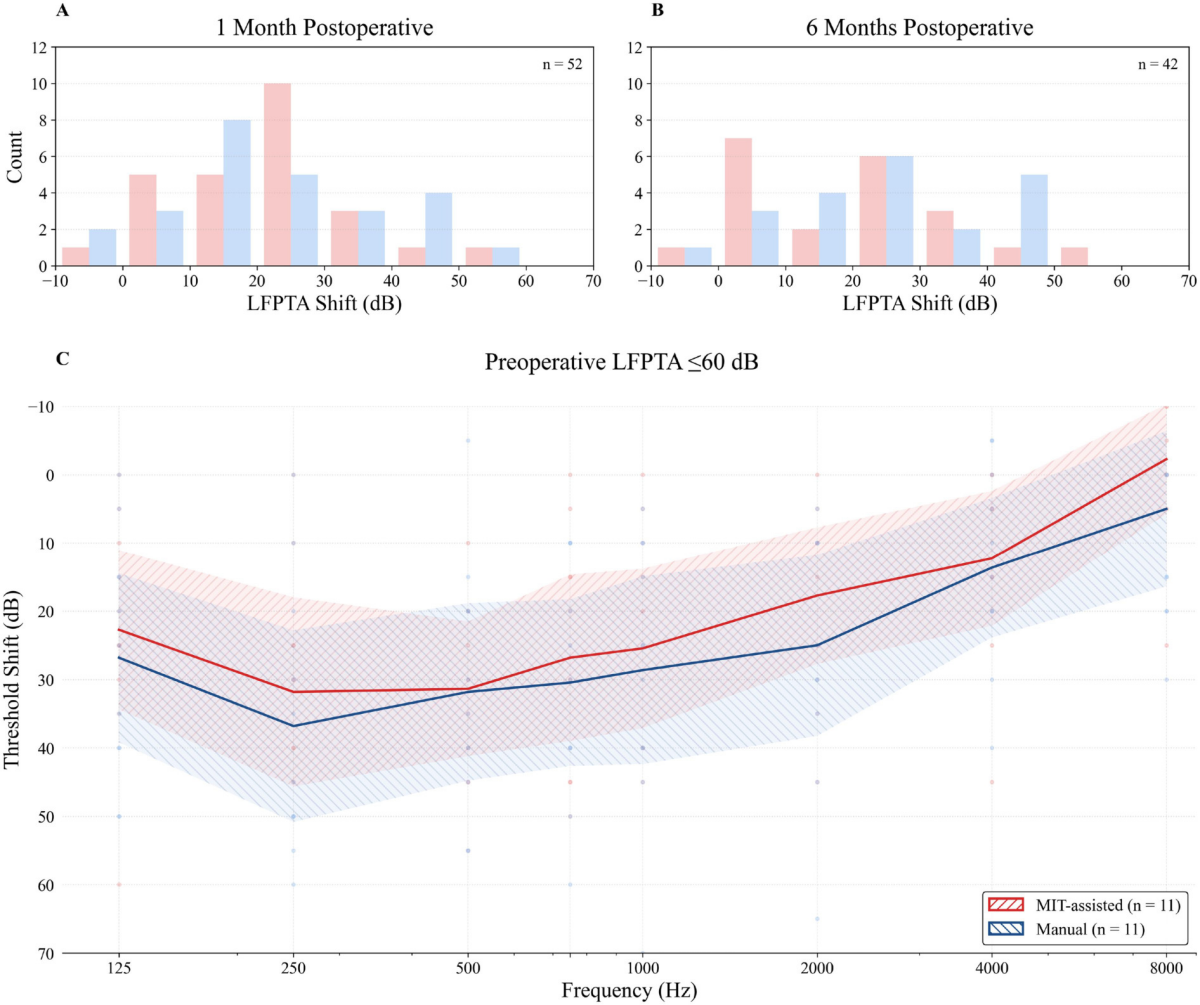
Histograms illustrate the distribution of low-frequency pure-tone average (LFPTA; 125–500 Hz) threshold shifts at 1 month **(A)** and 6 months **(B)** postoperatively relative to preoperative values. MIT-assisted insertions are shown in red, manual insertions in blue. **(C)** Frequency-specific threshold shifts from preoperative to 1 month postoperative audiometry in patients with preserved low-frequency hearing (preoperative LFPTA ≤60dB). Individual data points are displayed as small dots. Solid lines indicate group means, and shaded areas represent 95% CI. Positive values indicate hearing loss. Only patients with available postoperative follow-up at the respective time points were included.

As shown in Figure 2, the largest difference in low-frequency hearing preservation between cohorts was observed in patients with the best preoperative hearing (LFPTA ≤60dB). In this subgroup, the median shift at 1 month postoperatively was −26.7dB in the MIT-assisted cohort (n=11) compared with −36.7dB in the manual cohort (n=11). While the difference was statistically not significant (p=0.65), the absolute median difference of 10.0dB corresponded to a negligible effect size (Cliff’s δ=0.12). Figure 3 shows histograms of threshold shifts at 1 month (Panel A) and 6 months (Panel B), indicating a tendency toward reduced hearing loss in the MIT-assisted cohort. Frequency-specific threshold shifts across 125–8,000 Hz (Panel C) further supported improved preservation in this subgroup, particularly at lower frequencies.

When patients were grouped more broadly into two subgroups based on preoperative LFPTA better or worse than 80dB HL (49), the differences between cohorts disappeared. Using this criterion, 18 patients in the MIT group and 16 in the control group were classified as having preserved hearing. Median threshold shifts were comparable: 25.8dB (IQR=10.4) in the MIT group and 28.3dB (IQR=26.7) in the control group (p=0.817).

**Figure 4:**
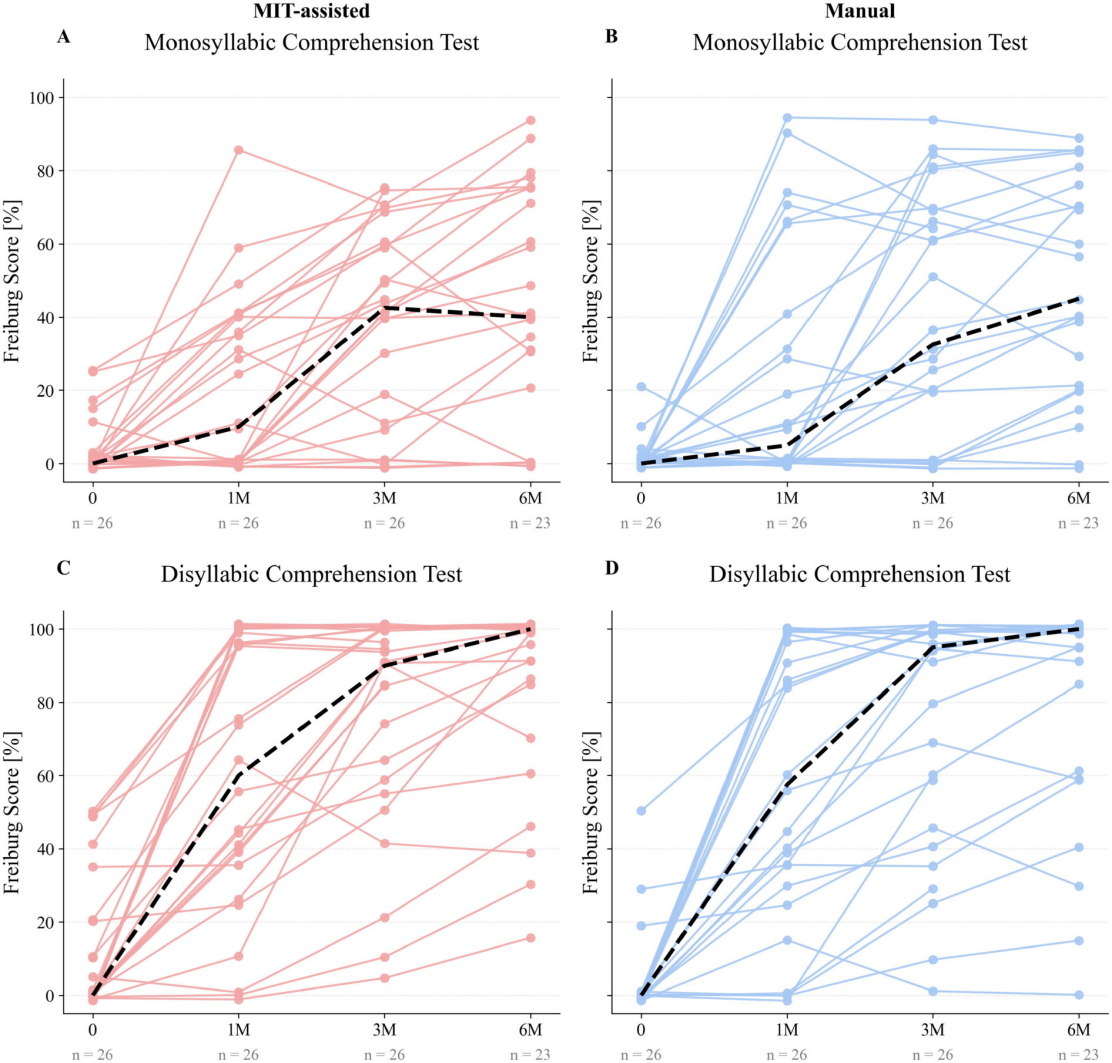
Paired line plots illustrate individual Freiburg speech test scores before electrode array implantation (0) and at 1, 3, and 6 months postoperatively (1M, 3M, 6M) for both MIT-assisted **(A, C)** and manual **(B, D)** cohorts. Panels **A–B** display monosyllabic comprehension scores, while panels **C–D** present disyllabic comprehension scores. Each trajectory represents one patient’s performance over time. Dashed black lines indicate the median score at each time point within the respective cohort. Speech comprehension scores are reported as percentages.

For speech comprehension, both the MIT-assisted and manual cohorts demonstrated similar overall improvement over time (Figure 4). For disyllabic comprehension, median scores increased from 0% preoperatively to approximately 60% at 1 month and reached 100% by 6 months in both groups, with slightly higher intermediate values in the MIT-assisted cohort. Monosyllabic comprehension improved more gradually, from 0% at baseline to 45% at 6 months, again following comparable trajectories between groups. As expected, owing to the greater difficulty of the monosyllabic test, inter-individual variability was higher; however, overall trends remained similar across cohorts.

### 3.3 Subjective speech comprehension outcomes

Due to the recent integration of standardized PROM collection into routine clinical practice, sample sizes differed between the MIT-assisted and manual cohorts, particularly for the preoperative assessments. Consequently, direct comparisons must be interpreted with caution, and formal statistical testing was not considered meaningful.

Preoperatively, the MIT-assisted cohort reported lower subjective ratings in most subscales. Postoperatively, however, this cohort showed greater score increases compared with the manual group. Overall, the MIT-assisted cohort demonstrated equal or higher mean scores in most domains, with particularly pronounced postoperative gains in “Speech in speech” comprehension, where the manual cohort exhibited only modest improvement. The only exception was the “localisation” domain, in which the manual group outperformed the MIT-assisted group.

**Figure 5:**
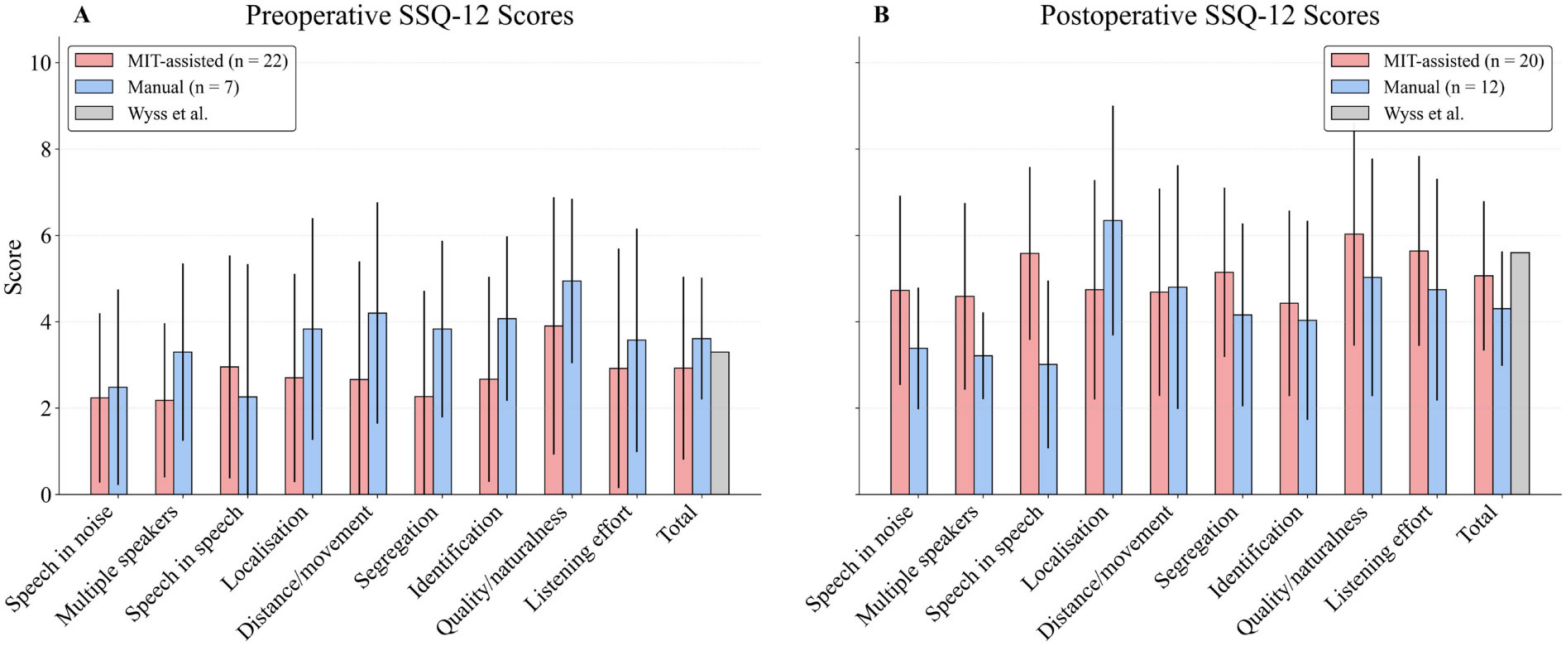
Bar plots display mean postoperative scores (± standard deviation) across the 9 domains and the total score of the Speech, Spatial, and Qualities of Hearing Scale (SSQ12), scaled from 0 to 10. Patients were grouped by surgical approach (MIT-assisted vs. Manual). As standardized questionnaire-based follow-up was only introduced later in clinical routine, the available dataset for both the MIT-assisted and manual groups is limited to cases collected since its implementation. A preoperative **(A)** and 12-month postoperative **(B)** benchmark score reported by Wyss et al. (50) is included for reference in the “Total” domain.

At 6 months postoperatively, the total score was 5.0 in the MIT-assisted cohort and 4.6 in the manual cohort. For comparison, Wyss et al. (50) reported a 12-month benchmark score of 5.6, which is higher than in our data and likely attributable to the shorter follow-up period in this study (50) (Figure 5).

## 4 Discussion

With the growing number of cochlear implant candidates presenting with measurable residual hearing prior to surgery, hearing preservation has become an increasingly important goal of cochlear implantation. As manual electrode insertion is subject to well-documented limitations, MIT systems have attracted increasing interest. These systems take over the electrode insertion into the cochlea, where slow, continuous, and controlled advancement is key.

In model studies using pressure and force sensors, MIT systems have demonstrated a clear advantage. However, the extent to which this benefit translates into real-world clinical settings remains debated. Early clinical studies have shown that postoperative deterioration of residual hearing thresholds can also occur in MIT-assisted patients (34, 51). This deterioration tended to be slightly less pronounced than with manual insertions (26). With the present study, we expand on these findings by reporting the first clinical application of the OTOARM/OTODRIVE system.

### 4.1 Surgical aspects

In our experience, the MIT system was seamlessly integrated into the clinical routine. While the total surgical time was slightly longer in the MIT group compared with manual insertion, the difference remained within clinically acceptable limits and was statistically insignificant. However, a distinction must be made: in many patients, alignment of the MIT tool and the electrode proceeded without problems or delays, whereas in some cases this step proved more challenging. This difficulty was partly related to the properties of Flex Electrodes, which, as their name implies, are highly flexible and do not typically align in a straight trajectory toward the round window opening. If the electrode tip deviated inferiorly from the round window, procedural delays could occur because the electrode required reorientation. Whether these additional manipulations are relevant for hearing preservation cannot be determined at this point.

The angular insertion depth (AID) between the two groups did not differ, and our data demonstrated that deep insertions, with median AID values of $573.8^{\circ }$, were achievable using the MIT system and are in accordance with previously published reports (31, 52, 53). Likewise, the distance from electrode contact 12 to the round window was comparable between groups, with an IQR of $19.6^{\circ }$ for the MIT-assisted cohort and an IQR of $18.6^{\circ }$ for the manual cohort, indicating that use of the MIT system did not compromise surgical visualization or increase the rate of incomplete insertions. Other clinical reports demonstrate that MIT-assisted insertions achieve high placement precision through reproducibly low and consistent insertion forces, while preserving intraoperative electrode control (24, 30).

An additional observation was that, with a table-mounted MIT system, the electrode did not remain entirely stationary when the system was at rest or during insertion, but exhibited a pulse-synchronous movement, which in some patients was visually apparent. The effect of this patient-related motion on intracochlear pressure and force dynamics remains unclear.

### 4.2 Hearing preservation outcomes

In patients with substantial preoperative residual hearing, slightly better postoperative low-frequency pure-tone hearing thresholds were observed following MIT-assisted insertions compared with manual insertions. However, this difference disappeared when the grouping criterion was broadened. On a macroscopic level, none of our patients (MIT-assisted or manual group) showed signs of scalar translocation in the postoperative CT scan. Similar findings have been reported by other authors, who also described not only improved functional residual hearing preservation, but also structural preservation, with fewer macroscopically detectable electrode translocations (33, 41).

While test bench studies have clearly demonstrated the benefits of MIT systems, it remains unclear to what extent these effects translate into the clinical setting and long-term outcomes. It is important to emphasize that cochlear implantation comprises more than electrode insertion alone. Previous studies have shown that other surgical factors can exert a decisive influence on residual hearing. For instance, cochlear access with a drill has been associated with substantial noise exposure (54). In addition, contamination of the perilymphatic space with blood or bone dust, as well as perilymph leakage through the round window, may compromise inner ear function (12, 13, 55). Moreover, electrode implantation does not end with the insertion phase. Evidence from both test bench experiments and clinical studies indicates that subsequent stabilization of the electrode within the facial recess and mastoid can generate considerable pressure fluctuations, which may be transmitted into the cochlea and result in measurable changes in electrophysiological recordings (21, 31, 56, 57).

Non-surgical factors may also contribute to the deterioration of residual acoustic function. Inflammatory responses to the intracochlear foreign body can lead to fibrosis or even ossification, thereby reducing residual function (58, 59). Finally, the underlying inner ear disease may progress independently of implantation (60, 61). For these reasons, the present data are insufficient to determine whether MIT systems provide a statistically significant improvement in residual hearing preservation.

With respect to speech comprehension, no clear advantage was observed for the MIT-assisted group in our cohort. The underlying expectation was that a gentler implantation might better preserve the neural interface and thereby improve performance scores. However, other research groups have likewise reported similar findings (26, 42, 43).

### 4.3 Subjective outcomes

From our perspective, subjective measures are at least as important as standardized audiometric tests, as they capture aspects of hearing performance that are essential for everyday communication but are not fully represented in audiometric assessments. Ultimately, improvements in functional and structural hearing preservation should also be reflected in enhanced subjective hearing perception. For this purpose, we used a questionnaire that is widely accepted and applied internationally. Given the data gaps in the manual implantation group, these findings must be interpreted with caution. Our intention was to provide an initial foundation on which future publications can build. In our figures, the MIT-assisted group started from lower baseline values but demonstrated greater improvements in the majority of subscales. Whether these differences represent genuine effects or are merely a consequence of the asymmetric data structure remains a question that can only be fully answered with a larger patient cohort.

### 4.4 Currently available MIT systems

Three primary Motorized Insertion Tool (MIT) systems are currently available for cochlear implantation: the table-mounted Otodrive (MED-EL/CASCINATION), the self-supporting Robotol (Collin Medical), and the patient-mounted IotaSOFT (iotaMotion). These systems are conceptually similar but differ in several key aspects. The IotaSOFT system consists of single-use components that cannot be reused, whereas the Otodrive and Robotol systems are reusable and supplied with sterile drapes. This results in distinct cost structures regarding initial acquisition and recurring expenses. For reusable systems, sterile covers are required and should be applied as closely as possible to the robotic arm to avoid restricting visualization. All three systems allow adjustment of the insertion angle in three dimensions to accommodate individual anatomical conditions. Mechanically, patient-mounted systems provide relative stabilization by moving synchronously with the patient, an approach that may mitigate the previously described pulse-synchronous motion artifacts more effectively than externally fixed-reference systems. Finally, all systems involve a learning curve; however, determining the optimal operational parameters for their use remains an important subject for future investigation.

### 4.5 Limitations

Several limitations must be acknowledged. First, the cohort size was small, which limited statistical power. The definition of our cohort size was based on the numbers of a comparable study design using another MIT product (Iotamotion, USA) (43). A post-hoc power analysis of our data indicated that approximately 56 patients per group would be required to detect a clinically relevant difference in low-frequency hearing preservation with sufficient confidence. Second, the retrospective design of the manual insertion cohort introduced potential selection bias and restricted control over confounding variables. Third, PROM data were incomplete in the manual cohort, precluding meaningful statistical comparisons for subjective outcomes. In addition, a potential bias might have occurred, as patients in the MIT-assisted cohort were aware that a motorized tool was used, which may have influenced subjective questionnaire responses through expectation bias. Future research should therefore employ a blinded, randomized design with larger sample sizes, extended follow-up, and complete PROM collection to more reliably evaluate the potential benefits of MIT systems for both objective and patient-reported outcomes.

## 5 Conclusion

In summary, our study demonstrated that integration of MIT systems into the clinical routine was feasible and did not compromise surgical workflow or electrode positioning. A tendency toward improved preservation of residual hearing was observed in patients with favorable preoperative hearing, although no clear advantage in speech comprehension was detected. Subjective measures suggested potential benefits, but the limited sample size and incomplete data precluded firm conclusions. Larger, blinded, and randomized studies with comprehensive PROM collection are required to clarify the clinical value of MIT systems for both objective and patient-reported outcomes.

## Data Availability

The raw data supporting the conclusions of this article will be made available by the authors upon request for academic, non-commercial purposes.
